# Usefulness of mirror image processing software for creating images of expected appearance after blepharoptosis surgery

**DOI:** 10.1007/s10792-020-01671-3

**Published:** 2020-12-04

**Authors:** Yuki Mawatari, Takahiro Kawaji, Hirohiko Kakizaki, Aric Vaidya, Yasuhiro Takahashi

**Affiliations:** 1 Mawatari Oculoplastic Clinic, 8-2 Suido-cho, Chuo-ku, Kumamoto, 860-0844 Japan; 2 Igo Ophthalmic Clinic, Kagoshima, Japan; 3 Sato Eye and Internal Medicine Clinic, Kumamoto, Japan; 4 Department of Oculoplastic, Orbital and Lacrimal Surgery, Aichi Medical University Hospital, Nagakute, Aichi Japan

**Keywords:** Blepharoptosis, Surgery, Mirror image, Simulation

## Abstract

**Purpose:**

This study aims to evaluate the usefulness of creating images of expected appearance after blepharoptosis surgery using mirror image processing software.

**Methods:**

This prospective, observational study included 60 sides from 30 patients with bilateral aponeurotic blepharoptosis who underwent levator resection or aponeurotic repair on both sides. Before surgery, facial photographs were taken after the eyelid on one side was lifted with a curved hook. The mirror images were created from these photographs and were merged with the original photographs for making the whole facial images, which were shown to each patient at the preoperative counseling. At 1 month postoperatively, we asked patients about the usefulness of the mirror images to predict the postoperative appearance at the preoperative counseling and the similarity between the expected and the resultant postoperative appearance using questionnaires. Margin reflex distance-1, eyebrow height, and pretarsal skin height measured on predictive images were compared with those measured at 1 month postoperatively.

**Results:**

Twenty-nine patients (96.7%) favorably responded to the usefulness of the mirror images to predict the postoperative appearance, and twenty-five patients (83.3%) accepted the similarity between the expected appearance and the actual postoperative appearance. The predictive images showed significantly lower margin reflex distance-1, higher eyebrow height, and higher pretarsal skin height than the postoperative results (*P *< 0.001).

**Conclusion:**

The creation of expected postoperative images was a useful simulation procedure before blepharoptosis surgery for patients to predict the probable postoperative appearance.

**Supplementary material:**

The online version contains supplementary material available at (10.1007/s10792-020-01671-3).

## Introduction

A postoperative change in the appearance is a major concern for patients before undergoing blepharoptosis surgery. The presentation of an expected postoperative appearance at the time of preoperative counseling can eliminate the patients’ anxiety. The presentation of pre- and postoperative face photos that were taken in other patients is helpful for clearly understanding the expected change. However, simulation of postoperative appearance in the patient’s own face can provide better imaginability for the patients. Elevation of the eyelid using a curved hook is a conventional simulation technique to create an expected postoperative appearance. However, it is quite hard to imagine the whole predictive appearance from face photos alone taken when the upper eyelid of only one side is elevated. On the other hand, bilateral eyelid lift using a curved hook is also difficult to be controlled.

Previously, we had reported the utility of a method for making images of predictive appearance after levator resection using image processing software (Adobe Photoshop^®^, San Jose, CA, US) [1]. However, this method was complicated and time-consuming. Additionally, in elderly patients and those with narrow eyelid fissures, the actual postoperative appearance was somewhat different from the predicted one, possibly resulting from an enlargement of the cornea which occurs during image processing.

In order to eliminate such defects, we developed a new method of making the whole facial images of predictive appearance after blepharoptosis surgery using the mirror image processing software. Herein, we evaluated the efficacy of this method in patients who underwent bilateral blepharoptosis surgery.

## Materials and methods

### Study design and patients

This prospective, observational study included 60 sides from 30 Japanese patients (13 males and 17 females; mean age, 69.4 years; age range, 42–84 years) with bilateral aponeurotic blepharoptosis who underwent levator resection or aponeurotic repair on both sides at Igo Ophthalmic Clinic between July 2019 and January 2020. Patients with poor levator function or patients in whom clear images could not be created were excluded from the study.

### Surgical procedures

Surgical procedures were selected according to the severity of blepharoptosis. We performed levator resection in moderate-to-severe cases (18 patients) and aponeurotic repair in mild cases (12 patients). After a subcutaneous and conjunctival forniceal injection of local anesthesia using 2% lidocaine with 1:80,000 epinephrine, the skin was incised along the planned new eyelid crease with a laser blade. The eyelid was fixed with an eyelid clamp. The levator aponeurosis was detached from the tarsal surface and then from Müller’s muscle for aponeurotic repair. In cases of levator resection, the levator aponeurosis and Müller’s muscle complex were dissected together from the palpebral conjunctiva. After removal of the clamp, the levator aponeurosis or levator complex was advanced onto the tarsus with 6-0 Nylon^®^ mattress sutures (Mani Co. Ltd. Inc., Tochigi, Japan). Intraoperative quantification was performed, while the patient was in sitting position. In cases with eyelid fullness, the preaponeurotic fat pad was removed as needed. After removal of an excess skin, the eyelid crease was formed, and the skin was closed with 6-0 Nylon^®^ sutures.

### Image processing

**Fig. 1:**
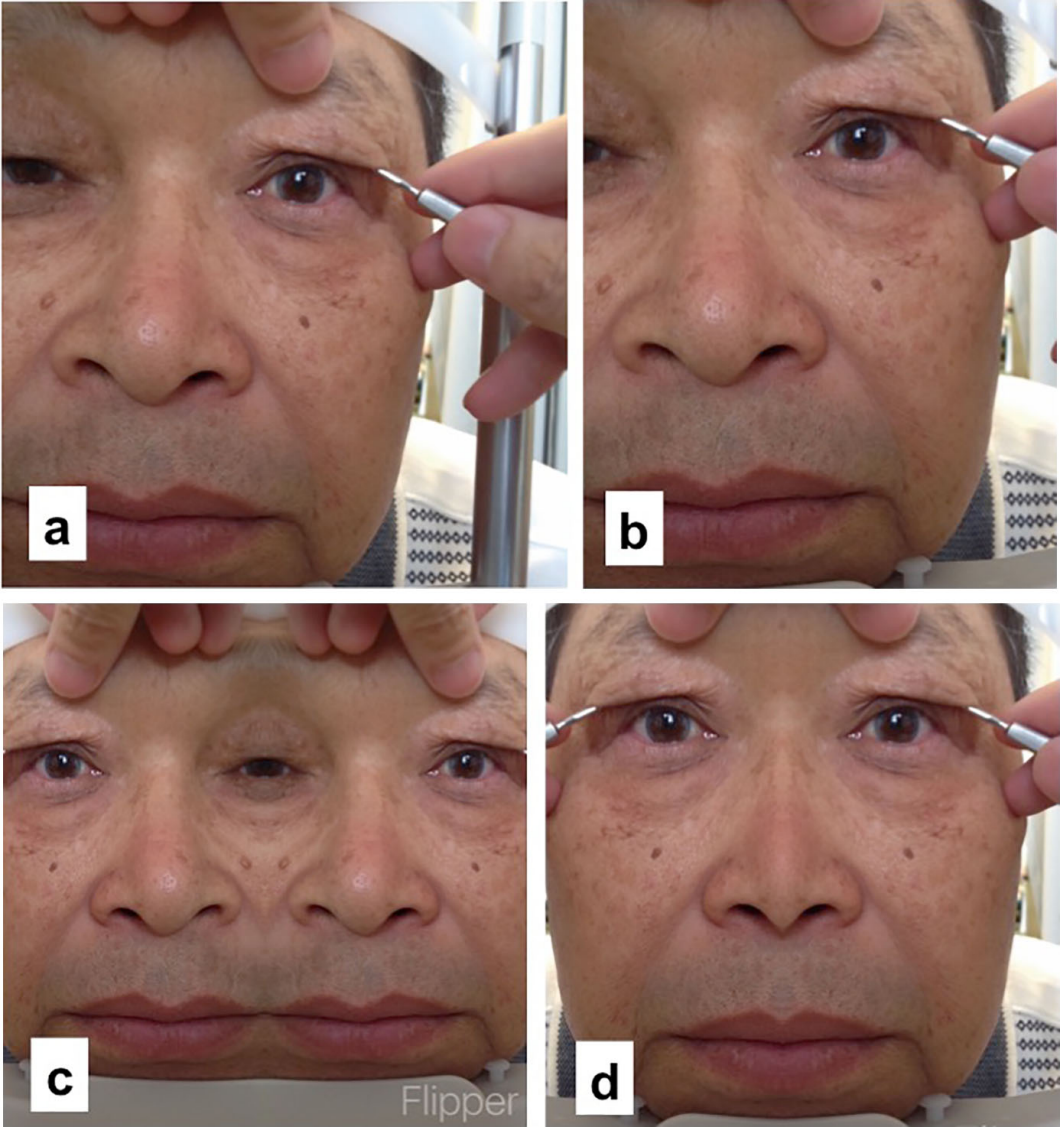
Image processing using the mirror image processing software. **a** Elevation of the eyelid skin on one side using a curved hook. **b** Fine-tuning of face tilt. **c** Creation of a mirror image. **d** Merging the original image with the mirror image
- *Step 3* A digital camera incorporated in iPhone or iPad was activated. When the right eyelid was lifted, photographs were taken so that the right eye was located at the center of the photograph. Face tilt on the photograph was fine-tuned (Fig. 1b).
- *Step 4* The mirror image processing software (Flipper, available with iPhone or iPad at apps.apple.com/jp/app/flipper…/id986445990) was launched, and then, a mirror image photograph was created automatically (Fig. 1c).
- *Step 5* The mirror image photograph was merged with the original photograph (Fig. 1d, see video).

The image processing procedures were as follows:

- *Step 1* The patient’s forehead and chin were fixed on the forehead and chin rest stands equipped in a slit lamp, respectively.
- *Step 2* The upper eyelid was lifted on one side using a curved hook until obtaining an adequate upper eyelid height and pretarsal show. Eyebrow position and extra skin were fine-tuned by hand (Fig. 1a).

### Outcome measures

#### Subjective outcomes

At 1 month postoperatively, we showed the mirror image and postoperative face photo to the patients and asked them to fill in a questionnaire, which included the following 2 questions to evaluate: (1) whether the processed images were useful to predict the postoperative appearance and (2) whether their actual postoperative appearances were similar to the expected images. Patients replied as either yes or no and put it into the document collection box.

#### Objective outcomes

Upper eyelid height before and after surgery was assessed by the margin reflex distance-1 (MRD-1), which is defined as the distance from the corneal reflex to the upper eyelid margin with the eyes in the primary position. Eyebrow height (EBH) was measured from the corneal reflex to the lowest eyebrow margin in the mid-pupillary plane. Pretarsal skin height (PSH) was measured from the double eyelid line to the eyelid margin in the mid-pupillary plane. Postoperative complications such as asymmetric upper eyelid position and under- or overcorrection of blepharoptosis were noted at 1 month postoperatively.

The measurements were performed on photographs taken before and 1 month after surgery using ImageJ software version 1.41 (National Institute of Health, Bethesda, MD, US). The corneal diameter was set to the standard scale of 11 mm.

### Statistical analysis

Statistical analyses were performed by JMP version 13.1 (SAS Institute Inc., Cary, NC, US). The significance of the differences between the pre- and postoperative measurement values and that between the postoperative measurement values and those measured on the predictive images were determined by the paired *t*-test. The value of *P *< 0.01 was considered to be statistically significant.

## Results

**Fig. 2:**
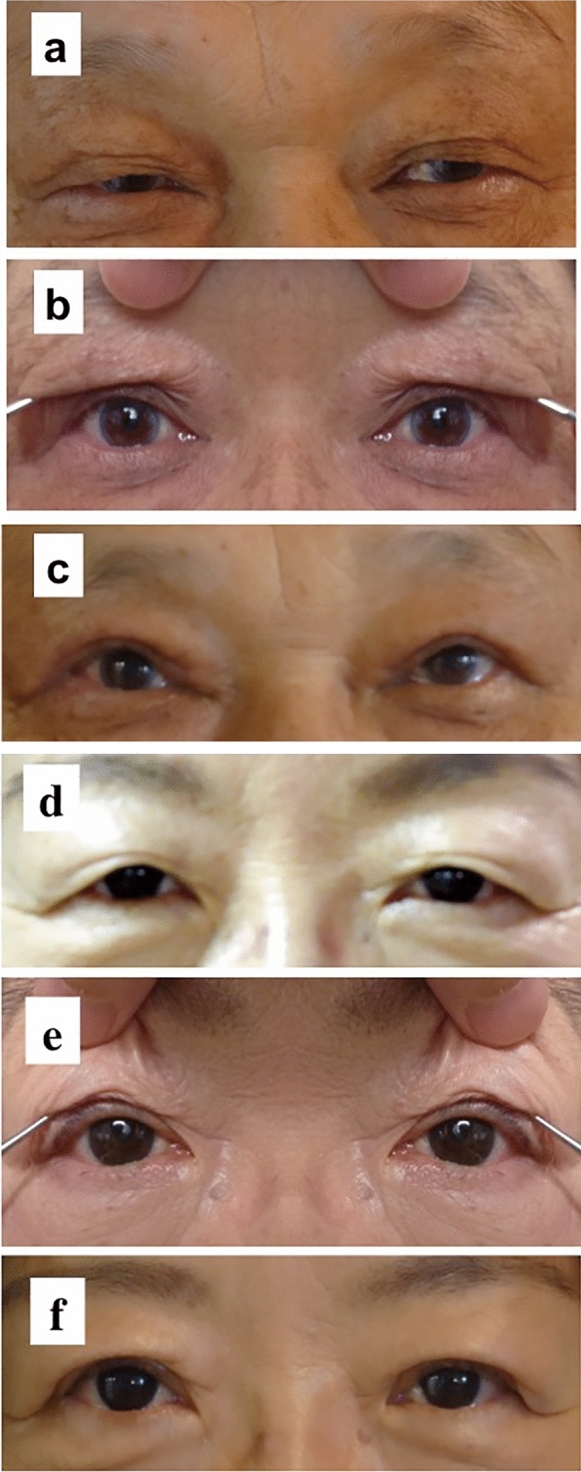
Presentation of two patients. **a**–**c** A 68-year-old man with aponeurotic blepharoptosis who underwent levator resection on both sides. **d**–**f** A 68-year-old woman with aponeurotic blepharoptosis who underwent aponeurotic repair on both sides

The representative 2 cases are shown in Fig. 2. Twenty-nine patients (96.7%) favorably responded to the usefulness of their mirror image to predict the postoperative appearance. Twenty-five patients (83.3%) answered that the actual postoperative appearance was similar to their predicted image. Three and two patients responded that the upper eyelid height was higher and lower than that on the predicted image, respectively.

The mean (± standard deviation) MRD-1 significantly increased from 0.6 ± 0.9 mm (range, − 1.0 to 2.0 mm) preoperatively to 3.4 ± 0.5 mm (range, 2.0 to 4.4 mm) at 1 month postoperatively (*P *< 0.001). The mean MRD-1 measured on the predictive photographic images was 2.5 ± 0.6 mm (range, 2.0 to 4.0 mm), which was significantly lower than the actual postoperative MRD-1 (*P *< 0.001).

The mean EBH significantly decreased from 22.0 ± 3.8 mm (range, 13.8 to 28.8 mm) preoperatively to 18.9 ± 3.3 mm (range, 12.3 to 24.6 mm) at 1 month postoperatively (*P *< 0.001). The mean EBH measured on predictive photographic images was 21.9 ± 3.8 mm (range, 12.7 to 30.6 mm), which was significantly higher than the actual postoperative EBH (*P *< 0.001).

The mean PSH significantly increased from 1.4 ± 2.0 mm (range, 0–5.9 mm) preoperatively to 2.9 ± 0.9 mm (range, 1.2–5.5 mm) at 1 month postoperatively (*P *< 0.001). The mean PSH measured on predictive photographic images was 3.6 ± 1.1 mm (range, 1.0–5.8 mm), which was significantly higher than the actual postoperative PSH (*P *< 0.001).

An incomplete eyelid closure of 1–2 mm occurred immediately after surgery in six sides (10.0%). However, this improved at 1 month postoperatively in all those patients. An asymmetric upper eyelid position following surgery was noted in two patients because of the under-correction of blepharoptosis on one side or the insufficient removal of the eyelid fat pad, such that these patients required additional surgeries.

## Discussion

The usefulness of images of predicted postoperative appearance created by the mirror image processing software was evaluated in this study. All except one patient, positively responded that the predictive images were useful to anticipate the postoperative appearance at the time of preoperative counseling. In addition, 83.3% of the patients accepted the resemblance between the predictive and the actual postoperative appearance. This simulation technique will be helpful to relieve the anxiety of the patients regarding the postoperative changes in their appearance before undergoing blepharoptosis surgery.

Our previously reported image processing method was complicated and time-consuming, and the cornea shown on the predicted images was enlarged during the image processing [1]. The mirror image processing software used in this study is freely available and can create the mirror images easily and quickly with the help of iPhone or iPad. Moreover, the corneal size does not change after image processing with this software. Although the mirror images can be created with Adobe Photoshop^®^ as well, this software is not freely available and the time required to transfer or edit images is relatively longer.

MRD-1, EBH, and PSH were different between the values measured on predictive images and the postoperative values. The MRD-1 value measured on the predictive photographic image was significantly lower than the actual postoperative value. The difference between the EBH values measured on the preoperative images and the postoperative values was approximately 3.1 mm, which was significant and was similar to those reported in the previous studies (2.4–4.5 mm) [2–4]. The PSH measured on the predictive photographic images was significantly higher compared to the actual postoperative PSH, and the postoperative PSH was closer to the PSH of normal Asians [5, 6]. In patients with an excess upper eyelid skin, the examiner may have unconsciously raised the upper eyelid using a curved hook to make an eyelid crease. Hence, in order to improve the accuracy of predictive photographic images in future, we need to adjust the MRD-1, EBH, and PSH while taking the predictive photographs. On the preoperative counseling, we need to intentionally set the target MRD-1 and PSH as 3–4 mm and 2–3 mm, respectively, and fix the forehead to the forehead rest more securely so as to prevent the contraction of the frontalis muscle.

As the face is essentially asymmetrical [7], patients may have some sense of incongruity for symmetrical photographic images. However, blepharoptosis surgery aims to lift not only the upper eyelids on both sides symmetrically in bilateral cases, but also the upper eyelid of one side to the same level of the other side in unilateral cases for improving the facial appearance. Therefore, although we need to explain the facial asymmetry, the creation of predictive postoperative images using the mirror image processing software is extremely useful at the time of preoperative counseling.

Asymmetric upper eyelid position following surgery was noted in 2 patients, and as mentioned above, the eyelid status was significantly different between the values measured on predictive images and the postoperative values. However, most patients may understand that the postoperative eyelid status does not completely correspond to the status on the predictive images. Patients may, therefore, be able to accept discrepancies between the predictive photographic images and the actual postoperative outcomes, resulting in favorable responses to our questionnaires.

The current study was limited by several factors. As we included only the patients of aponeurotic blepharoptosis with fair-to-good levator function, the procedure presented in this study may not be applied to patients with other types of blepharoptosis or with poor levator function. We did not include controls in whom the upper eyelid was elevated only on one side at the time of preoperative counseling. In addition, as the eyelid anatomy shows a racial difference, the results of our study performed in Japanese people may not be applicable for other races.

In conclusion, as creation of the predictive images using the mirror image processing software is simple and quick, this technique can be taken into daily practice for oculoplastic surgeons at the time of preoperative counseling.

## Supplementary Information

Below is the link to the electronic supplementary material.

Video: Image-processing using the mirror image-processing software.Supplementary material 1 (MP4 15835 kb)

## Data Availability

All data are included in this paper.
